# Mechanism of Epimedium intervention in heart failure based on network pharmacology and molecular docking technology

**DOI:** 10.1097/MD.0000000000032059

**Published:** 2022-11-25

**Authors:** Chen Boyang, Li Yuexing, Yan Yiping, Yu Haiyang, Zhao Lingjie, Guan Liancheng, Zhang Xufei, Zhao Jie, Chen Yunzhi

**Affiliations:** a School of Preclinical Medicine, Guizhou University of Traditional Chinese Medicine, Guiyang, Guizhou, China; b Second Affiliated Hospital, Guizhou University of Traditional Chinese Medicine, Guiyang, Guizhou, China.

**Keywords:** Epimedium, heart failure, molecular docking, molecular mechanism, molecular targets, network pharmacology

## Abstract

To analyze the pharmacological mechanism of Epimedium in regulating heart failure (HF) based on the network pharmacology method, and to provide a reference for the clinical application of Epimedium in treating HF. Obtaining the main active ingredients and their targets of Epimedium through TCMSP (Traditional Chinese Medicine Systems Pharmacology Database and Analysis Platform) database. Access to major HF targets through Genecards, OMIM, PharmGKB, Therapeutic Target Database, Drug Bank database. Protein interaction analysis using String platform and construction of PPI network. Subsequently, Cytoscape software was used to construct the “Epimedium active ingredient-heart failure target” network. Finally, the molecular docking is verified through the Systems Dock Web Site. The core active ingredients of Epimedium to regulate HF are quercetin, luteolin, kaempferol, etc. The core targets are JUN, MYC, TP53, HIF1A, ESR1, RELA, MAPK1, etc. Molecular docking validation showed better binding activity of the major targets of HF to the core components of Epimedium. The biological pathways that Epimedium regulates HF mainly act on lipid and atherosclerotic pathways, PI3K-Akt signaling pathway, and chemoattractant-receptor activation. And its molecular functions are mainly DNA-binding transcription factor binding, RNA polymerase II-specific DNA-binding transcription factor binding, and neurotransmitter receptor activity. This study reveals the multi-component, multi-target and multi-pathway mechanism of action of Epimedium in regulating mental failure, and provides a basis for the clinical development and utilization of Epimedium to intervene in HF.

## 1. Introduction

Heart failure (HF) is a disease caused by various causes of structural changes or dysfunction of the heart, filling of the ventricles or impaired blood expulsion.^[1]^ In developed countries, the prevalence of known HF is generally estimated at 1% to 2% of the general adult population and increasing as the world’s population expands and ages.^[2]^ As a traditional kidney tonic, Epimedium has the function of “tonifying the liver and kidney, strengthening the muscles and bones, and dispelling wind and dampness.” Modern pharmacological studies have shown that Epimedium has good intervention effects on cardiovascular diseases under the pharmacological effects of cardiovascular protection, immune regulation and anti-inflammation. In HF models, epimedium based formulations inhibited myocardial transforming growth factor-β1 expression and alleviated HF by reversing ventricular hypertrophy. However, the role of the active ingredients in the formula and the intervention mechanism of HF has not been fully clarified.

Traditional Chinese medicine network pharmacology prioritizes disease-related genes by drug-gene-disease co-module association to predict the target spectrum and pharmacological effects of effective compounds in Chinese herbal medicines.^[3]^ In this study, we investigated the material basis and mechanism of action of Epimedium in the treatment of HF using network pharmacology. Molecular docking of some key targets will lay the foundation for the future development and application of Epimedium in the treatment of HF.

## 2. Materials and methods

### 2.1. Screening of active ingredients of Epimedium. Searching for Epimedium

Compound components using the Traditional Chinese Medicine System Pharmacology Database (TCMSP).^[4]^ Preliminary screening was then performed to obtain effective active compounds and their protein targets based on 2 properties: oral availability  ≥ 30% and drug-like properties ≥ 0.18.^[5]^ After screening, the protein targets are uniformly converted into corresponding genes in the Uniprot protein database.

### 2.2. HF targets selection

We searched for related genes by entering “heart failure” in Gene Cards, OMIM, PharmGKB, Therapeutic Target Database and Drug Bank, and obtained disease targets and Epimedium therapeutic targets. The common target was selected as the potential target of Epimedium for the treatment of HF.

### 2.3. Epimedium active ingredient-HF target Venn diagram and PPI diagram

The active ingredient-HF target of Epimedium was plotted with Venny to create a Venn diagram. The intersecting targets are then submitted to the STRING database to build a protein protein interaction network (PPI), where the interaction information of each protein is automatically scored, with higher scores indicating higher confidence in protein interactions. For the input target, the biological species was set to “Homo sapiens” and the minimum interaction threshold was set to “highest confidence.” The obtained high-confidence protein interactions data were used to construct PPI.

### 2.4. Active ingredients of Epimedium-HF targets gene ontology (GO) and Kyoto encyclopedia of genes and genomes (KEGG) enrichment analysis

GO and KEGG enrichment analysis of the active ingredients of Epimedium-HF targets using R Cluster Profiler package.^[6]^ Three parameters of GO function, Biological Process, Molecular Function and Cellular Component, were selected for gene enrichment analysis, screening *P* < .01, and results were visualized using R.

The network topology parameters of the effective components and targets are analyzed using CytoScape’s built-in Network Analyzer, including connectivity, betweenness centrality (BC) and closeness centrality (CC), etc. The selection criterion is to choose the Degree that is greater than the median value of all points, and finally the targets that meet the median values of BC and CC as the core targets. The core targets and the main active ingredients are determined based on the network topology parameters, and the 2 are used for molecular docking.

### 2.5. Molecular docking of active ingredients of Epimedium with key targets in HF

AutoDock Vina was used as the molecular docking software. For further validation, the key active ingredients of Epimedium with the top 3 “Degree” values were selected for molecular docking with the top 3 “Degree” ranked targets in the PPI network. The 2D structures of the key active ingredients were downloaded using the PubChem database and saved in SDF file format. ChemBio3D software was used to retouch to SDF file format and optimize its 3D structure and save as mol2 format. The crystal structures of the targets were downloaded from the PDB database and processed using PyMOL software for removal of water molecules, hydrogenation, etc. The 3D structures of key active ingredients of Epimedium were then converted to pdbqt file format using AutoDock Tools with the target crystal structure, and molecular docking was performed using Autodock Vina software.^[7]^ The binding activity of the key active ingredient to the target was evaluated by binding, and the results of compound and protein docking were observed and analyzed using PyMOL software.

## 3. Results

### 3.1. Epimedium active ingredients

The active ingredients and targets of Epimedium were initially extracted as 130 species by searching the TCMSP database, and 23 major active ingredients were obtained after screening, including quercetin, luteolin, kaempferol, etc. Other active ingredients of Epimedium (Table 1). There were 1620 targets for the action of Epimedium, and 105 targets were obtained after combining and deleting duplicate values.

**Table 1:** The 23 main active ingredients of Epimedium.

Mol ID	Molecule names	OB(%)	DL
MOL001510	24-epicampesterol	37.58	0.71
MOL001645	Linoleyl acetate	42.1	0.2
MOL001771	poriferast-5-en-3beta-ol	36.91	0.75
MOL001792	DFV	32.76	0.18
MOL003044	Chryseriol	35.85	0.27
MOL003542	8-Isopentenyl-kaempferol	38.04	0.39
MOL000359	sitosterol	36.91	0.75
MOL000422	kaempferol	41.88	0.24
MOL004367	olivil	62.23	0.41
MOL004373	Anhydroicaritin	45.41	0.44
MOL004380	C-Homoerythrinan, 1,6-didehydro-3,15,16-trimethoxy-, (3.beta.)-	39.14	0.49
MOL004382	Yinyanghuo A	56.96	0.77
MOL004384	Yinyanghuo C	45.67	0.5
MOL004386	Yinyanghuo E	51.63	0.55
MOL004388	6-hydroxy-11,12-dimethoxy-2,2-dimethyl-1,8-dioxo-2,3,4,8-tetrahydro-1H-isochromeno[3,4-h]isoquinolin-2-ium	60.64	0.66
MOL004391	8-(3-methylbut-2-enyl)-2-phenyl-chromone	48.54	0.25
MOL004394	Anhydroicaritin-3-O-alpha-L-rhamnoside	41.58	0.61
MOL004396	1,2-bis(4-hydroxy-3-methoxyphenyl)propan-1,3-diol	52.31	0.22
MOL004425	Icariin	41.58	0.61
MOL004427	Icariside A7	31.91	0.86
MOL000006	luteolin	36.16	0.25
MOL000622	Magnograndiolide	63.71	0.19
MOL000098	quercetin	46.43	0.28

DL = drug-like properties, OB = oral availability.

### 3.2. Epimedium active ingredient target-HF target intersection and construction of PPI network

**Figure 1.**
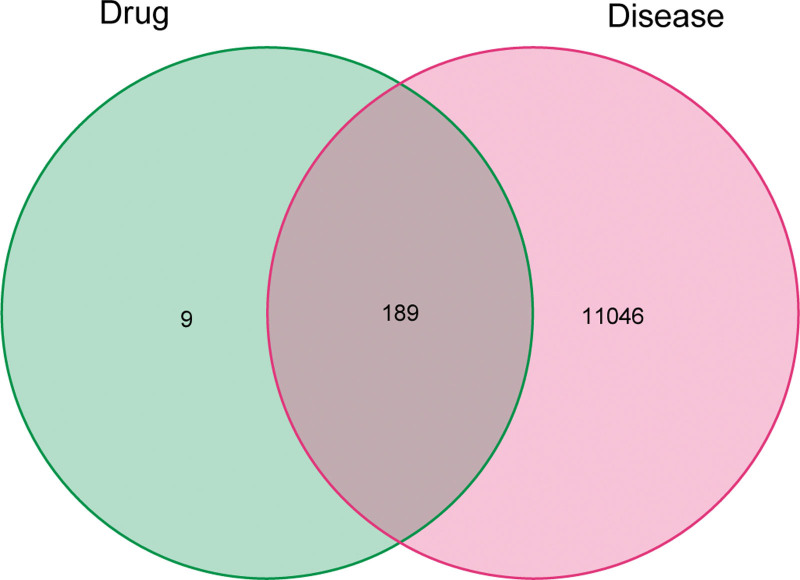
Venn diagram.

**Figure 2.**
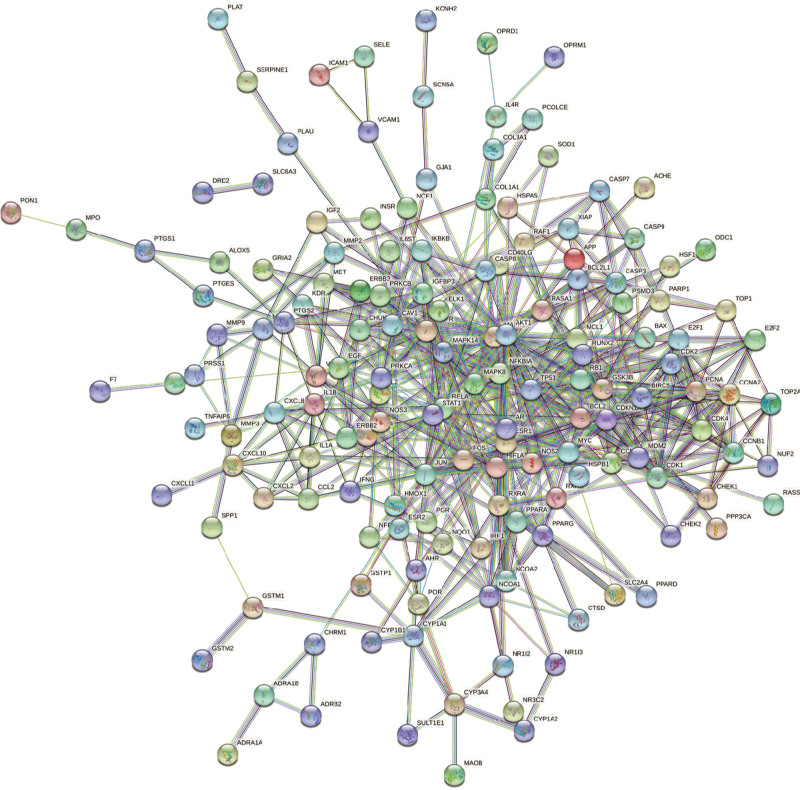
PPI network. PPI = protein protein interaction.

Venn diagram of the active ingredient targets of Epimedium (Fig. 1). After taking the intersection of the active ingredient targets of Epimedium and the HF targets, 189 common targets were obtained. The targets were submitted to the STRING platform, and the protein interaction threshold was set to “highest confidence” to obtain the Epimedium-HF target PPI network (Fig. 2).

### 3.3. Active ingredients of Epimedium-HF targets GO functional enrichment and KEGG pathway analysis

**Figure 3.**
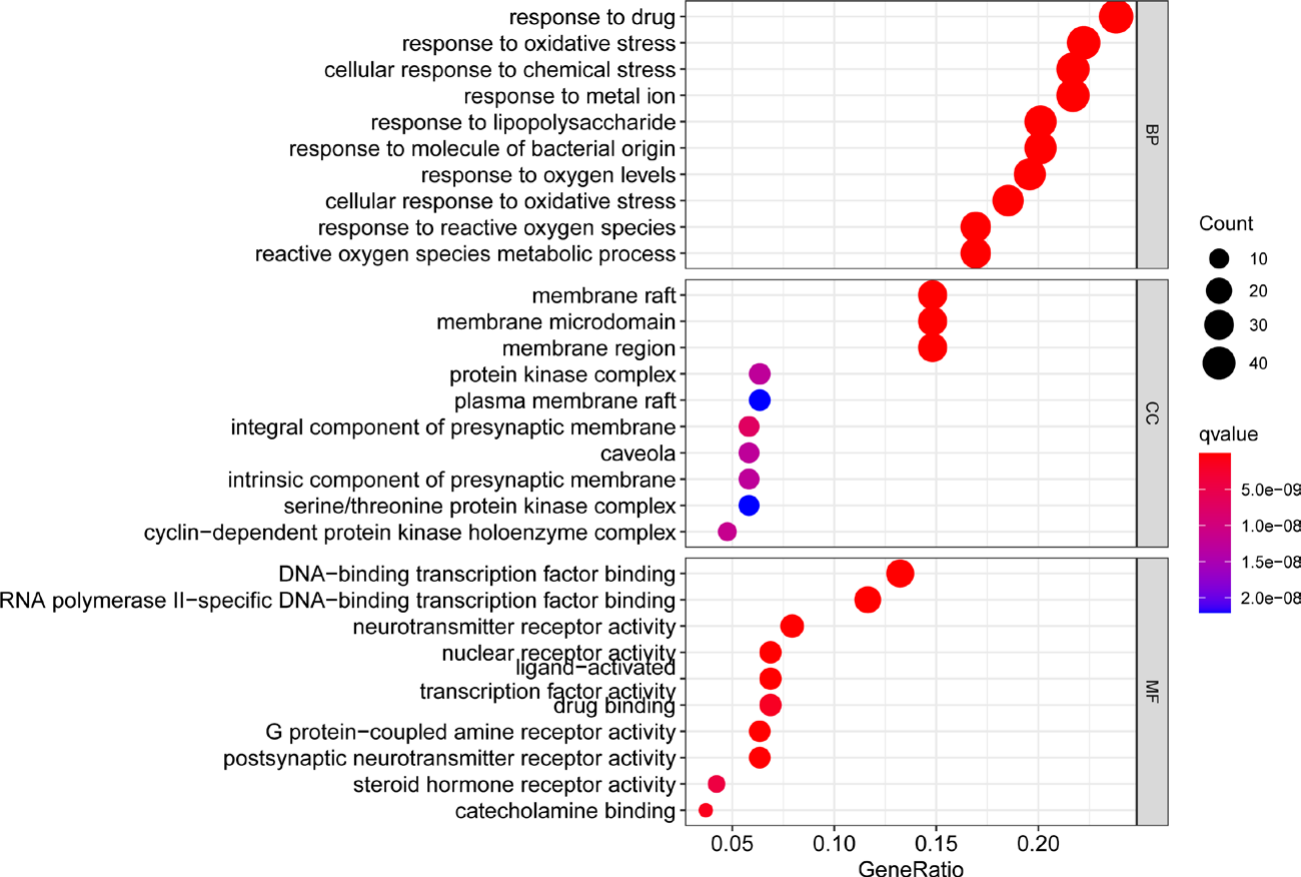
GO enrichment analysis of Epimedium to regulate HF. GO = gene ontology, HF = heart failure.

**Figure 4.**
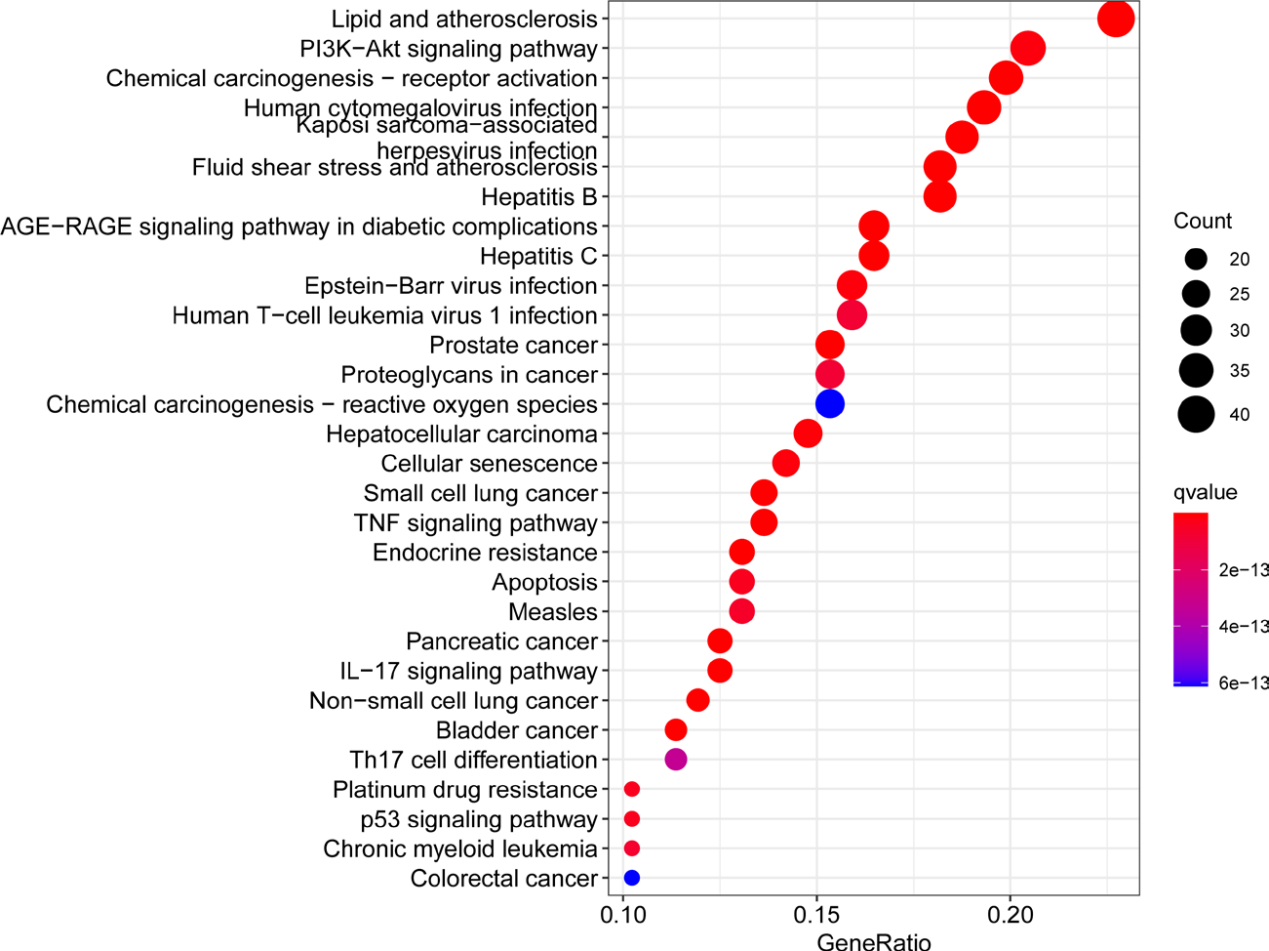
KEGG enrichment analysis of Epimedium to regulate HF. HF = heart failure, KEGG = Kyoto Encyclopedia of Genes and Genomes.

The R Cluster Profiler software package was applied to analyze the signaling pathways of Epimedium’s targets related to the regulation of HF. GO biofunctional enrichment analysis of 189 common targets showed that Epimedium is mainly involved in biological processes, including drug response, oxidative stress, and metal ion response. The molecular functions in which Epimedium is mainly involved include DNA-binding transcription factor binding, RNA polymerase II-specific DNA-binding transcription factor binding, and neurotransmitter receptor activity. The cellular components that Epimedium participate in include membrane raft, membrane microdomain and membrane region. KEGG pathway analysis showed that the main target enrichment pathways of Epimedium to regulate HF are Lipid and atherosclerosis, PI3K-Akt signaling pathway, Chemical carcinogenesis-receptor activation, etc (Figs. 3 and 4).

**Figure 5.**
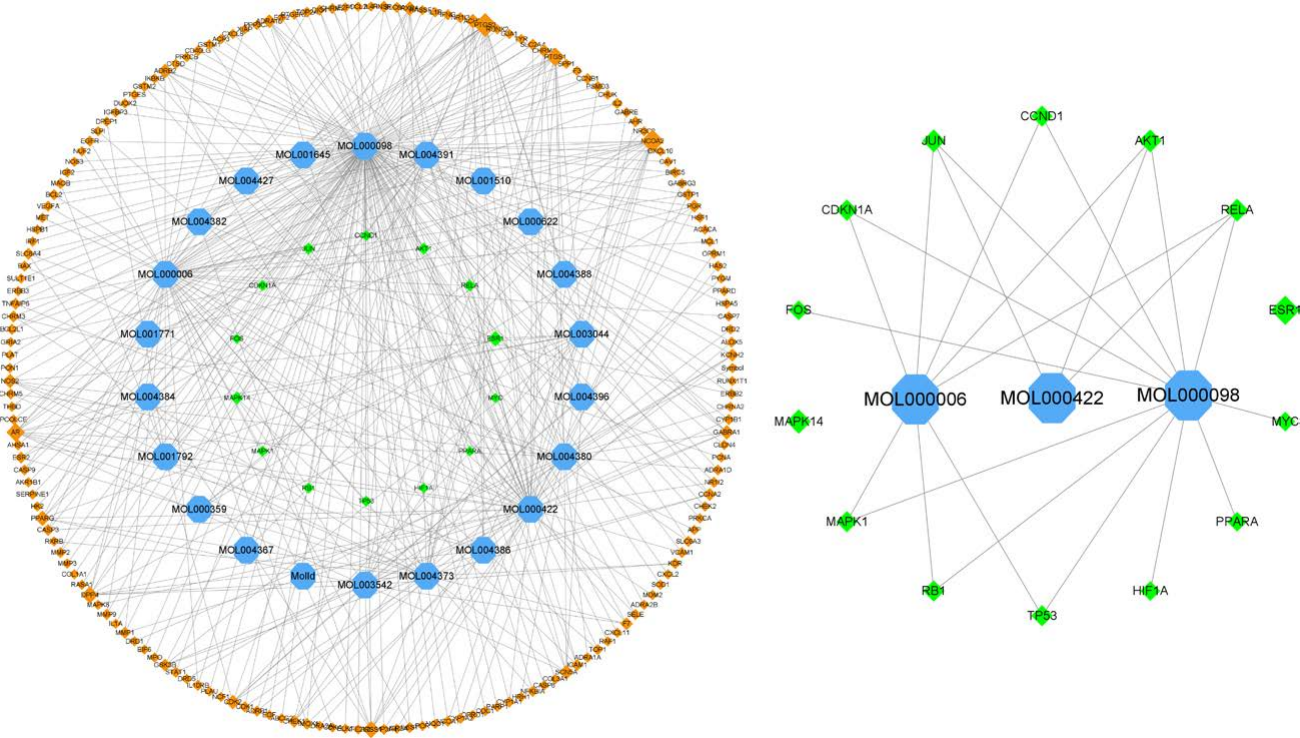
Epimedium active ingredients-HF targets network map. HF = heart failure.

The network topological parameters of Epimedium for HF were analyzed by Network Analyzer to obtain the core components. CytoScape network analysis showed that quercetin connectivity was 134, BC 0.70 and CC 0.62, presumably quercetin is the main component of Epimedium in regulating HF. Luteolin connectivity was 52, BC 0.16, CC 0.41; kaempferol connectivity was 51, BC 0.13, CC 0.41, both of which have important roles in the regulation of HF by Epimedium. The remaining active ingredient network analysis values (Table 2). Epimedium active ingredients-HF targets network map (Fig. 5).

**Table 2:** Active ingredients in Epimedium to regulate heart failure.

Ingredients	BC	CC	Degree
quercetin	0.693379	0.616519	134
luteolin	0.156386	0.412229	52
kaempferol	0.132334	0.413861	51
C-Homoerythrinan, 1,6-didehydro-3,15,16-trimethoxy-, (3.beta.)-	0.144463	0.376577	30
Anhydroicaritin	0.057938	0.376577	28
8-(3-methylbut-2-enyl)-2-phenyl-chromone	0.044570	0.369912	25
8-Isopentenyl-kaempferol	0.031353	0.368607	20
Chryseriol	0.012232	0.358491	14
Yinyanghuo C	0.004103	0.352445	9
DFV	0.003426	0.351261	8
1,2-bis(4-hydroxy-3-methoxyphenyl)propan-1,3-diol	0.012949	0.346600	8
Yinyanghuo E	0.001178	0.350084	7
Yinyanghuo A	0.001024	0.347754	5
Linoleyl acetate	0.000470	0.346600	4
sitosterol	0.009818	0.322034	3
6-hydroxy-11,12-dimethoxy-2,2-dimethyl-1,8-dioxo-2,3,4,8-tetrahydro-1H-isochromeno[3,4-h]isoquinolin-2-ium	0.000111	0.344316	2
Magnograndiolide	0.000111	0.344316	2
24-epicampesterol	0.000013	0.339837	2
olivil	0.000249	0.321045	2
poriferast-5-en-3beta-ol	0.000249	0.321045	2
Icariside A7	0.009569	0.314286	2
MolId	0.000000	1.000000	1

BC = betweenness centrality, CC = closeness centrality.

### 3.4. Molecular docking of active ingredients of Epimedium with key targets in HF

**Figure 6.**
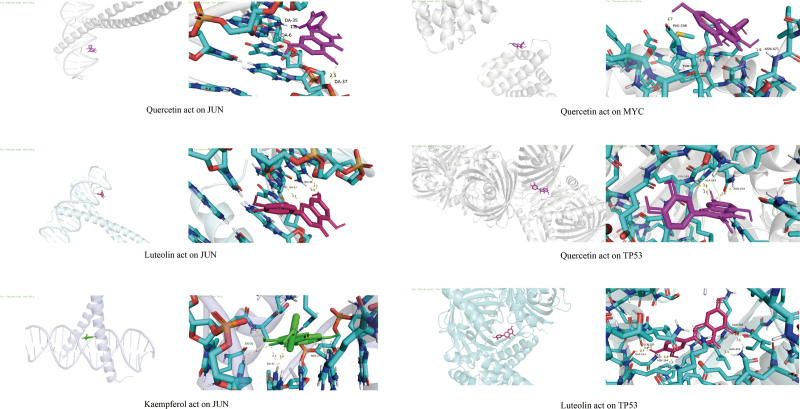
Molecular docking map of key components of Epimedium and HF targets. HF = heart failure.

The Autodock Vina software was used to molecularly dock the 3 main ligand molecule compounds of Epimedium quercetin, luteolin, and kaempferol with the top 3 core targets of HF Degree values and the remaining target information (Table 3). The docking results were visualized in PyMOL software and the optimal structural type was selected according to the binding energy, docking results (Table 4 and Fig. 6). The results showed that quercetin could form hydrogen bonds with JUN via DA-6, DA-35 and DA-37, with MYC via ASN-471, THR-507 and PHE-509, and with TP53 via ALA-163, ASN-164 and GLN-183.Luteolin can form hydrogen bonds with JUN via DA-37 and DG-38, and with TP53 via ALA-163, ASN-164, ASN-345, GLN-183, GLN-248, and ARG-342.kaempferol can form hydrogen bonds with JUN via DA-9, DA-31, DG-30, and ARG-270. The binding energies of the key components to the core targets were all below -5 kcal-mol-1, suggesting that the 3 key components have high bioaffinity to the core targets of HF and possess significant pharmacodynamic activity.

**Table 3:** HF targets information.

Targets	BC	CC	Degree
JUN	10.25714286	1	26
MYC	8.507142857	0.928571429	24
TP53	4.473809524	0.866666667	22
RELA	3.835714286	0.8125	20
MAPK1	4.55	0.8125	20
HIF1A	3.438095238	0.8125	20
ESR1	2.34047619	0.8125	20
MAPK14	2.685714286	0.764705882	18
CCND1	3.233333333	0.764705882	18
RB1	4.54047619	0.722222222	16
FOS	0.785714286	0.722222222	16
AKT1	1.9	0.722222222	16
CDKN1A	0.869047619	0.684210526	14
PPARA	0.583333333	0.619047619	10

BC = betweenness centrality, CC = closeness centrality, HF = heart failure.

**Table 4:** Predicting the binding ability of active ingredients of Epimedium to the targets site.

Targets	Compound	Binding affinity (kcal/mol)	Binding sites
JUN	quercetin	−8.7	DA-6, DA-35, DA-37
JUN	luteolin	−8.8	DA-37, DG-38
JUN	kaempferol	−8.6	DA-9, DG-30, DA-31, ARG-270
MYC	quercetin	−6.2	ASN-471, THR-507, PHE-509
TP53	quercetin	−8.3	ALA-163, ASN-164, GLN-183
TP53	luteolin	−8.7	ALA-163, ASN-164, GLN-183, GLN-248, ARG-342, ASN-345

The above results indicate that quercetin, luteolin and kaempferol, the 3 key components, have better binding activity to the core targets of HF, suggesting that quercetin, luteolin and kaempferol may act on the above core targets to play the function of regulating HF.

## 4. Discussion

HF is the ultimate destination of cardiovascular disease, and its occurrence is mainly related to inflammation, immunity, oxidative stress, vascular endothelial dysfunction, neurotransmitters and other factors.^[8]^ Epimedium has immunomodulatory, anti-inflammatory and antioxidant effects and is widely used in cardiovascular diseases. However, the main components and mechanism of action of Epimedium in regulating HF are unknown. In this study, we constructed Epimedium active ingredients-HF targets network, Epimedium target proteins PPI network for HF treatment, and key targets molecular docking through network pharmacology to analyze the possible action mechanism of Epimedium in regulating HF in a deeper and systematic way.

According to the TCMSP, 23 active ingredients of Epimedium regulating HF were screened, and then the drug activity and HF target proteins were matched to each other to obtain the intersection. 22 active ingredients corresponding to 189 common target proteins were obtained. Finally, the top 3 Degree values were taken, quercetin, luteolin and kaempferol were identified as the core components of HF regulation, and the rest of the components played a synergistic role in regulating HF. Quercetin, a major dietary flavonoid, has anti-angiogenic, anti-inflammatory, antioxidant and apoptotic biological effects that attenuate tumor proliferation and progression. Luteolin is a flavonoid widely found in a variety of plants, including herbs, with powerful anti-inflammatory activity. It can fight against a variety of malignancies by inhibiting the proliferation of tumor cells, protecting them from oncogenic stimuli and activating cell cycle blocking. Blocking tumor development in vivo and in vivo by inducing apoptosis through different signaling pathways.^[9]^ Kaempferol is a major flavonoid sapogenin with antibacterial, anti-inflammatory, antioxidant, antitumor, cardioprotective, neuroprotective and antidiabetic effects.^[10]^

Predicting drug targets reveals important information about the relationship between drugs and molecular mechanisms of action, which is important for facilitating drug development. A total of 189 intersecting targets were screened by constructing a PPI network map of Epimedium intervention targets in HF. The targets with the strongest correlation of Degree values were JUN, RELA, MYC, HIF1A, TP53, MAPK1, and ESR1. JUN family proteins play important roles in the regulation of inflammatory responses, oxidative stress, and cell survival, proliferation and differentiation.^[11]^ It was shown that angiotensin II-induced cardiac remodeling was associated with increased p-c-Jun/p-Jun-B translocation and expression.^[12]^ In addition, AP-1(JUN) was significantly expressed in congestive HF due to ischemic or dilated cardiomyopathy.^[13,14]^ RELA(p65) is a transcriptionally active subunit of activated nuclear factor-κB (NF-κB) dimer containing RELA and p50, which has the role of promoting gene expression in various biological processes such as immune response, inflammation, development, apoptosis and tumorigenesis.^[15]^ Inhibition of p65 improves cardiac function, reduces ventricular mass, and decreases myocardial cell apoptosis.^[16]^ MYC is a multifunctional transcription factor that regulates the expression of numerous genes in cell growth, proliferation and apoptosis and metabolic pathways.^[17]^ High MYC expression drives tumor initiation, progression and maintenance, and is associated with aggressive cancer and poor prognosis.^[18]^ Study found that MYC expression was significantly increased in bone marrow samples from patients with HF compared to normal subjects.^[19]^ HIF transcription factors are the main regulators of the cellular response to hypoxia. HIF-1α is expressed in a variety of cancer and immune cells and promotes tumor development, invasion and metastasis by inducing angiogenesis, as well as regulating cellular metabolism in the hypoxic tumor microenvironment.^[20]^ In addition, HIF-1α is involved in the inflammatory response. HF is a chronic inflammatory process that is closely associated with hypoxia. It was found that HIF-1α mRNA expression was increased in failing hearts during disease exacerbation in rats with HF.^[21]^ TP 53 is a tumor suppressor that regulates cell cycle, apoptosis, and DNA repair by regulating gene expression in response to cellular stress. Under physiological conditions, TP53 is functionally active to maintain cardiac structure, but elevated TP53 expression can lead to myocardial fibrosis, myocardial hypertrophy, myocyte apoptosis, non-myocyte proliferation, and left ventricular dilation leading to cardiac dysfunction.^[22]^ Therefore, inhibition of TP53 may serve as one of the effective therapeutic strategies to prevent the transition from cardiac hypertrophy to HF. MAPK 1 promotes cell proliferation and differentiation through cell cycle processes while inhibiting apoptosis.^[23]^ Study shows that MAPK 1 promotes cardiomyocyte proliferation.^[24]^ In addition to its important role in human reproduction, ESR also has a significant impact on adipose tissue and metabolism, bone, cardiovascular, brain and immunity.^[25]^ ESR1 KO mice were found to have reduced myocardial hypertrophy.^[26]^ Common ESR1 polymorphisms are significantly associated with age-related changes in left ventricular structure. Mechanisms of susceptibility to adverse cardiac left ventricular remodeling as we age may help identify new therapeutic targets for the prevention of HF.^[27]^ The above literature research shows that the target screening of Epimedium is consistent with the results of a large number of pharmacological experiments, and the relationship between the pharmacological components and the target action can be further investigated at a later stage based on the predicted results.

The results of GO functional enrichment analysis suggest that Epimedium affects HF through the action of related biological processes, molecular functions, and cellular composition. The results of KEGG pathway enrichment analysis showed that Epimedium achieves HF regulation through related disease pathways and signaling pathways, such as lipid and atherosclerosis, PI3K/Akt signaling pathway, and Chemical carcinogenesis-receptor activation. Atherosclerosis is a chronic inflammatory condition, and inflammation is an important causative mechanism of HF. Inflammatory factor TNF-α induces cardiomyocyte hypertrophy, activates metalloproteinases, inhibits metalloproteinase inhibitors, and leads to myocardial fibrosis.^[28,29]^ NF-κB, which is closely related to inflammation, mediates inflammation, apoptosis and extracellular matrix remodeling after activation by TNF-α.^[30]^ PI3K/Akt signaling pathway plays an important role in the regulation of cardiomyocyte growth, myocardial angiogenesis, and cell death.^[31]^ Meanwhile, activation of this pathway can induce monocyte chemotaxis, macrophage migration, increased intracellular lipid accumulation, neovascularization and lesion dysfunction, all of which are closely related to atherosclerotic plaque formation.^[32]^ In HF-related studies, the literature suggests that drugs can reduce reactive oxygen species production and myocardial apoptosis as well as reduce myocardial hypertrophy by modulating the PI3K/Akt signaling pathway.^[33]^ miRNA expression is altered by environmental chemicals such as metals, organic pollutants, cigarette smoke, pesticides and carcinogenic drugs.^[34]^ For example, arsenic regulates the PI3K/AKT/mTOR pathway through 2 different mechanisms to induce cell proliferation and apoptosis.^[35]^ Long-term exposure to arsenic promotes cell proliferation and tumor transformation, and induces non-carcinogenic diseases such as cardiovascular disease.^[36]^ Therefore, it is hypothesized that the active ingredients in Epimedium may affect the downstream genes and pathways through PI3K/Akt signaling pathway, thus regulating the development of HF.

The molecular docking results showed that quercetin had the lowest docking energy with JUN, suggesting that quercetin, as the core component of Epimedium, plays a major regulatory role in HF. The combination of Luteolin and JUN is more advantageous, and to a certain extent, it synergizes with quercetin, kaempferol and other components to enhance the regulatory effect on JUN.

A review of domestic and international studies showed that Epimedium down-regulates PI3K and Akt phosphorylation levels by inhibiting the activation of PI3K/Akt signaling pathway, reducing the proliferation of cancer cells and their ability to invade and migrate, and accelerating cancer cell apoptosis. Epimedium significantly inhibits MYC expression and alleviates cardiac dysfunction. Icariside down-regulates HIF-1α and other expressions to promote the proliferation and migration of endothelial progenitor cells, thereby repairing damaged endothelium and promoting.^[37,38]^ Icariside reduces NF-κB p65 (RELA) nuclear translocation by inhibiting or reducing NF-κB signaling pathway,^[39]^ reducing the levels of inflammatory factors such as TNF-α, IL-1β and IL-6, interfering with myocardial ischemia-reperfusion injury and inhibiting the progression of aortic atherosclerosis may be the potential mechanisms of Epimedium to regulate HF. Epimedium II, the active ingredient of Epimedium, down-regulates the activity of oxidative stress-related proteins such as JUN and inhibits the expression of TP53, thereby reducing cardiomyocyte apoptosis and improving left ventricular remodeling.^[40]^

In addition, many components in Epimedium can regulate HF gene expression, such as quercetin, luteolin, and kaempferol. It was shown that quercetin significantly inhibited the expression levels of RELA, JUN mRNA and protein in lipopolysaccharide-induced mouse sarcolemma and exerted anti-inflammatory and antioxidant effects.^[41]^ Quercetin regulates c-myc to inhibit apoptosis in cardiomyocytes during cardiac hypertrophy.^[42]^ Quercetin attenuates sodium nitrite-induced hypoxia in rats by inhibiting the HIF-1α pathway has a protective effect on cardiac tissue.^[43]^ Vanadium quercetin complex inhibits cancer progression by regulating pathways such as TP53 and downregulates cell proliferation associated with increased apoptotic events.^[44]^ Quercetin may protect against cardiovascular disease by regulating MAPK signaling cascade.^[45]^ In addition, quercetin protects against myocardial ischemia-reperfusion injury by reducing oxidative stress, inhibiting the inflammatory cascade, apoptosis and PI3K/Akt pathway.^[46]^ Luteolin inhibits AP-1 DNA binding activity to exert anti-inflammatory and other effects.^[9]^ Luteolin inhibits cell proliferation by reducing TNF-α-induced RELA expression.^[47]^ Luteolin inhibits PI3K/Akt and MAPK pathways to regulate TP53 expression for anti-proliferative and pro-apoptotic effects.^[48]^ Luteolin regulates endothelial dysfunction through multiple pathways such as MAPK and PI3K/Akt, inhibits apoptosis, and thus improves cardiomyocyte contractile function.^[49,50]^ Kaempferol activates anti-inflammatory activity by blocking lipopolysaccharide-induced signaling pathways such as AP-1 in macrophages, thereby inhibiting the release of inflammatory mediators.^[51]^ Anti-inflammatory effect of kaempferol against lipopolysaccharide-induced mastitis is associated with inhibition of NF-κB p65.^[52]^ Kaempferol inhibits MAPK pathway and regulates oxidative stress to reduce apoptosis and improve cardiac function.^[53]^ Kaempferol promotes PI3K and Akt phosphorylation and downregulates PI3K/Akt pathway to induce apoptosis in cancer cells, which has an important role in cardiovascular and other diseases.^[54]^

## 5. Conclusions

In summary, MYC, HIF-1α, NF-κB, JUN, TP53 and other genes as downstream regulators of PI3K/Akt signaling pathway, it is hypothesized that Epimedium may regulate the expression of the above genes through PI3K/Akt signaling pathway, and then regulate the downstream signaling pathways of PI3K/Akt pathway, such as HIF-1 signaling pathway and NF-κB signaling pathway, to exert intervention in HF. In this study, the mechanism of Epimedium’s intervention in HF was elucidated from the regulatory effects of Epimedium on the genes of JUN, MYC and TP53 and their protein expressions, which provided a basis for the clinical application of Epimedium to regulate HF. Due to the limitations of the network pharmacology methodology and the complexity of the chemical reactions occurring during the decoction of herbal components. The chemical composition of Epimedium decoction and the metabolites of its active ingredients in vivo were not included in the analysis of this study. In future, further screening on this basis will be followed by cellular or animal experiments to validate the main regulatory targets of Epimedium.

## Author contributions

Chen Boyang wrote this paper. Li Yuexing and Yan Yiping collected data. Yu Haiyang, Zhao Lingjie, Guan Liancheng, Zhang Xufei and Zhao Jie analyzed and organized data, Chen Yunzhi reviewed the manuscript.

**Data curation:** Yu Haiyang, Zhao Lingjie, Guan Liancheng, Zhang Xufei, Zhao Jie.

**Funding acquisition:** Chen Yunzhi.

**Writing – original draft:** Chen Boyang, Li Yuexing, Yan Yiping.
